# The association between multisite musculoskeletal pain and cardiac autonomic modulation during work, leisure and sleep – a cross-sectional study

**DOI:** 10.1186/s12891-018-2312-3

**Published:** 2018-11-20

**Authors:** Tatiana de Oliveira Sato, David M. Hallman, Jesper Kristiansen, Andreas Holtermann

**Affiliations:** 1 Physical Therapy Department, Federal University of São Carlos (UFSCar), Rodovia Washington Luís, km 235, São Carlos, SP 13565-905 Brazil; 2 Centre for Musculoskeletal Research, Department of Occupational and Public Health Sciences, University of Gävle, 801-76 Gävle, SE Sweden; 3 National Research Centre for the Working Environment (NRCWE), Lersø Parkallé 105, 2100 Copenhagen Ø, DK Denmark; 4 Department of Sports Science and Clinical Biomechanics, University of Southern Denmark, Odense, Denmark

**Keywords:** Heart rate variability, Autonomic nervous system, Chronic pain, Physical activity

## Abstract

**Background:**

The prevention and rehabilitation of multisite musculoskeletal pain would benefit from studies aiming to understand its underlying mechanism. Autonomic imbalance is a suggested mechanism for multisite pain, but hardly been studied during normal daily living. Therefore, the aim of the study is to investigate the association between multisite musculoskeletal pain and cardiac autonomic modulation during work, leisure and sleep.

**Methods:**

This study is based on data from the “Danish Physical activity cohort with objective measurements” among 568 blue-collar workers. Pain intensity scales were dichotomized according to the median of each scale, and the number of pain sites was calculated. No site was regarded as the pain-free, one site was considered as single-site musculoskeletal pain and pain in two or more sites was regarded as multisite musculoskeletal pain. Heart rate variability (HRV) was measured by an electrocardiogram system (ActiHeart) and physical activity using accelerometers (Actigraph). Crude and adjusted linear mixed models were applied to investigate the association between groups and cardiac autonomic regulation during work, leisure and sleep.

**Results:**

There was no significant difference between groups and no significant interaction between groups and domains in the crude or adjusted models for any HRV index. Significant differences between domains were found in the crude and adjusted model for all indices, except SDNN; sleep time showed higher values than leisure and work time, except for LF and LF/HF, which were higher during work.

**Conclusion:**

This cross-sectional study showed that multisite musculoskeletal pain is not associated with imbalanced cardiac autonomic regulation during work, leisure and sleep time.

**Electronic supplementary material:**

The online version of this article (10.1186/s12891-018-2312-3) contains supplementary material, which is available to authorized users.

## Background

Chronic musculoskeletal pain has high prevalence and large consequences for the society [12, 34]. Although most studies focus on pain localized in a particular body region (single-site pain), such as low back pain or neck/shoulder pain [41], musculoskeletal pain usually occurs concurrently in several anatomical sites [4, 7]. This condition is called multisite pain [31] and has been shown to be associated with increased healthcare utilization, sick leave, early retirement, sickness and social welfare benefit [11, 13, 17].

In contrast to single-site pain, which is considered to be due to overload or insufficient use of a particular body region [7], multisite pain may be driven by more generalized mechanisms, such as an imbalance in autonomic cardiac modulation [42].

Autonomic cardiac modulation can be assessed by heart rate variability (HRV) reflecting parasympathetic and sympathetic regulation of beat-to-beat heart rate. Recent systematic reviews show moderate evidence supporting a decrease in parasympathetic modulation in chronic pain patients [20, 40]. However, we are aware of only one study (the Netherlands Study of Depression and Anxiety - NESDA) investigating the association between multisite musculoskeletal pain and HRV, finding no relationship between HRV and pain onset [14] or recovery [15]. However, more studies on the association between multisite musculoskeletal pain and HRV are required before any conclusion can be drawn.

For better prevention and rehabilitation of multisite musculoskeletal pain, it is important to understand the underlying mechanism [8], e.g., if it is related to an imbalanced autonomic regulation. It is also relevant to investigate large populations with a wide variation in the number of pain sites, and the variation of autonomic activity throughout daily living (not only artificial conditions). Moreover, because physical activity and body postures influence HRV in ambulatory recordings [2, 5, 33], it is important to use valid technical information of physical activity and body postures during the measurement of HRV. Also, a previous study of HRV in a working population showed differences between work and leisure time only for the sitting posture [35]. None of the previous studies on multisite musculoskeletal pain and HRV has taken all these factors into account. Thus, the aim of this study was to investigate the association between multisite musculoskeletal pain and cardiac autonomic modulation during work, leisure and sleep, and the interaction between multisite musculoskeletal pain and time domains.

## Methods

### Study population and exclusion criteria

This is a cross-sectional study based on data from “The Danish Physical activity cohort with objective measurements” (DPhacto) cohort, conducted on blue-collar workers recruited in the cleaning in public and private sector (i.e. hospitals, schools, municipalities, and private firms), manufacturing and production companies in metal, plastic, and food industries, and transportation (i.e. mail, and parcel service companies). The recruitment was performed in collaboration with a labor union and the data were collected between 2012 and 2013. The inclusion criteria were: to be allowed to participate during the paid working time, to be employed for more than 20 h per week and being between 18 and 65 years. Exclusion criteria were declining to sign the informed consent, pregnancy, fever on the testing day, and allergy to adhesives.

**Fig. 1:**
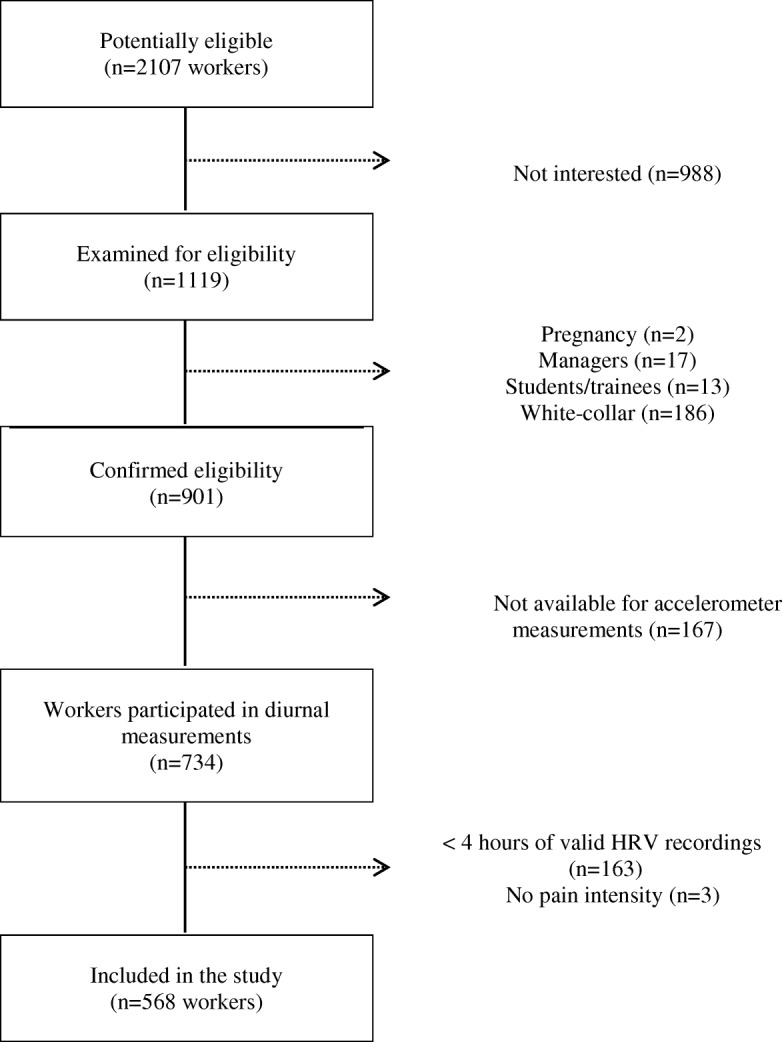
Flowchart of the study population

Among 2107 potentially eligible workers in the DPhacto cohort, objective measurement data from 759 blue-collar workers were available for analysis. Workers having less than 4 h of valid HRV recordings during work, leisure and sleep time (*n* = 163) and with no information about pain intensity (*n* = 3) were excluded, resulting in a final sample of 568 blue-collar workers (Fig. 1). The response rate was 63%. Detailed information about the DPhacto cohort can be found elsewhere [18].

### Multisite musculoskeletal pain

Musculoskeletal pain was assessed by modified questions from the validated Nordic Musculoskeletal Questionnaire [23] with questions on pain intensity in seven anatomical areas (neck/shoulders; elbows; hands/wrists; low back; hips; knees and feet/ankles) during the past three months using a scale from 0 (no pain) to 10 (worst pain). Pain intensity scales were dichotomized from the median, i.e. less than median = ‘no pain’, more than median = ‘pain’ [31]. The cut-off values were 3 points for neck/shoulder and low back, and 0 points for elbows, hands/wrists, hips, knees, feet/ankles. The elbows and hands/wrists were grouped to represent the upper limbs and the hips; knees and feet/ankles represented the lower limbs. The four regions were summed to provide information about the number of pain sites (0 = none to 4 = 4 pain sites). Pain in none of the regions was regarded as ‘pain-free’, one pain site was regarded as ‘single-site pain’, while the pain in two or more sites was defined as ‘multisite musculoskeletal pain’.

### Technical measurements of heart rate variability and physical activity

HRV was measured by the ActiHeart system (Camntech Ltd., Cambridge, UK) with electrocardiography sensitivity of 0.250 mV. The sensor was attached by a two-led configuration at the recommended position [3]. The analogue signal was band-pass filtered (10–35 Hz), sampled with a frequency of 128 Hz, and processed by a real-time QRS-detection algorithm to achieve a 1 ms time resolution of the RR intervals. Since data was collected during daily living conditions, respiratory rate was not controlled. Abnormal beats were removed using an automatic algorithm before analyzing HRV [22].

HRV data were analyzed using a robust method [37] from 5-min windows with less than 10% erroneous interbeat intervals (IBI). For time domain, the measures obtained were the standard deviation of R-R intervals (SDNN), which is a measure of overall variability; and the root mean square of successive differences of R-R intervals (RMSSD), a measure of beat-to-beat variability, which is related to the vagal modulation. For frequency domain, the low (LF, 0.04–0.15 Hz) and high frequency (HF, 0.15–0.40 Hz) components were analyzed, as well as the sympathovagal balance (LF/HF ratio). HF indicates the parasympathetic modulation of the cardiac rhythm, while LF is an indicator of both sympathetic and parasympathetic cardiac modulations [26, 28].

Physical activity and body posture were objectively measured using multiple accelerometers (ActiGraph GT3X+, Actigraph, Florida, USA). The accelerometers were attached to the thigh and upper back for several days, including work, leisure and sleep time. A diary was also filled out by the workers, including information about times getting up in the morning, starting and finishing work, and going to bed, as well as times of the reference position (upright stance) for calibration of the accelerometer records [18].

Physical activity and HRV data were processed using the Acti4 software (The National Research Centre for the Working Environment, Copenhagen, Denmark and BAuA, Berlin, Germany). The Acti4 software classifies different physical activities (walking, moving, cycling and running) and body postures (sitting, standing, and lying down) with high sensitivity and specificity [36]. The HRV indices obtained during 5-min non-overlap intervals in sitting posture at work and leisure were analyzed, as well as three periods with the lowest R-R intervals during nocturnal sleep without movement [16].

### Assessment of individual and occupational factors

A self-reported questionnaire was administered to the workers including age, gender, alcohol and tobacco use, medication prescription, job seniority, lifting and carrying during work. Height (cm) was measured using a scale (Seca, model 123) and weight (kg) was measured by a digital scale (Tanita model BC 418 MA). Body mass index (BMI) was calculated according to the formulae BMI = weight (kg)/height^2^ (m).

### Statistical analysis

Groups were compared for the characterization variables using one-way ANOVA and Tukey’s post hoc tests for continuous variables and Chi-square test for categorical variables. The HRV indices, except IBI, showed a non-normal distribution (Kolmogorov Smirnov test; see Additional file 1: Figures S1, S2 and S3). Thus a natural logarithm (ln) transformation was applied.

Linear mixed models with two fixed factors were applied to verify the association between groups (pain-free, single-site pain, multisite pain), domains (work, leisure, sleep), and interaction of groups and domains. Linear mixed models were chosen because it includes fixed and random effects and increase the study power including in the analysis subjects with missing data. Subject and intercept were included as random effects. The covariance type was unstructured, and the restricted maximum likelihood (REML) estimation method was chosen. When the interaction was significant, the mean difference (MD), standard error (SE) and *P* value for the pairwise comparison, based on estimated marginal means, were reported.

Crude and adjusted models were tested. In the adjusted model, age, sex, BMI, tobacco use, objectively measured moderate and vigorous physical activity (i.e. fast walking, running, stair climbing and cycling) at work and leisure, and sitting time at work and leisure were included as covariates since these factors may affect both multisite pain and HRV [1, 10, 19, 21, 29, 32]. Sensitivity analyses, excluding workers reporting prescribed medication in the last three months and considering the cut-off point of zero to dichotomize the pain intensity scales, were also performed. Stratified analyses were applied to explore possible effect modification of age (< 50 years; ≥50 years) and sex (male; female). All analyses were performed using SPSS software (version 24.0), and the significance level was set at 1% to control for type I error in multiple comparison tests.

## Results

About 44% of the sample was composed of females, the mean age was 45 years and mean BMI was 27 kg/m^2^. About 29% of the workers smoked daily or occasionally; 19% reported to use analgesic medication, 12% antihypertensive, 3% heart medication and 3% antidepressants. Most of the workers were from the manufacturing sector (72%), and 41% reported to carry and lift for at least half of the work time. The prevalence of single-site pain was 23% (CI 95%, 20–27%), and the prevalence of multisite pain was 63% (CI 95%, 58–66%). In both groups, the most affected body parts were knees (single-site pain: 32%; multisite pain: 67%), lower back (single-site pain: 13%; multisite pain: 65%) and neck/shoulder (single-site pain: 15%; multisite pain: 65%).

The three groups were similar in most of the sociodemographic variables, except for a larger proportion of lifting and carrying almost all the time to ¼ of the time in the multisite pain group. Pain intensity, the number of pain sites and the proportion of workers with prescribed analgesic medication were also higher in the multisite pain group compared with the pain-free and single-site pain groups. There were no differences between the groups for the other types of medication (Table 1).

**Table 1:** Characteristics of the blue-collar workers in DPhacto according to the pain groups. Continuous data are present as [mean (SD)], and frequencies are presented as [n (%)]

Characteristics	All (*n* = 568)	Pain-free (*n* = 74)	Single-site pain (*n* = 136)	Multisite pain (*n* = 358)	*P* value
Female	247 (43.5%)	25 (33.8%)	57 (41.9%)	165 (46.1%)	0.13
Age, years	45.3 (9.8)	46.8 (10.3)	45.0 (9.3)	45.1 (9.8)	0.39
Body mass index, kg/m^2^	27.3 (4.7)	27.2 (4.8)	26.9 (4.8)	27.5 (4.7)	0.43
Smokers	161 (29.1%)	18 (24.3%)	40 (31.0%)	103 (29.4%)	0.58
Alcohol, units/week	4.5 (5.9)	5.6 (7.2)	3.9 (4.7)	4.6 (6.0)	0.15
Use of medication	234 (41.2%)	25 (33.8%)	42 (30.9%)	167 (46.6%)	< 0.01
Antihypertensive	70 (12.3%)	9 (12.2%)	14 (10.3%)	47 (13.1%)	0.69
Heart	19 (3.3%)	3 (4.1%)	4 (2.9%)	12 (3.4%)	0.91
Antidepressants	18 (3.2%)	0 (0.0%)	2 (1.5%)	16 (4.5%)	0.06
Analgesic	108 (19.0%)	7 (9.5%)	14 (10.3%)	87 (24.3%)	< 0.01
Other	128 (22.5%)	14 (18.9%)	28 (20.6%)	86 (24.0%)	0.52
Occupational sector					0.44
Cleaning	109 (19.2%)	11 (14.9%)	31 (22.8%)	67 (18.7%)	
Manufacturing	410 (72.2%)	57 (77.0%)	97 (71.3%)	256 (71.5%)	
Transportation	49 (8.6%)	6 (8.1%)	8 (5.9%)	35 (9.8%)	
MVPA work time, h/day	1.3 (0.5)	1.3 (0.5)	1.3 (0.5)	1.3 (0.5)	0.95
MVPA leisure time, h/day	0.9 (0.3)	0.9 (0.4)	0.9 (0.4)	0.8 (0.3)	0.40
Sitting work time, h/day	2.5 (1.7)	2.5 (1.7)	2.5 (1.6)	2.5 (1.8)	0.95
Sitting leisure time, h/day	4.7 (1.3)	4.7 (1.5)	4.6 (1.3)	4.7 (1.3)	0.93
Seniority, months	13.4 (10.4)	12.9 (10.2)	13.1 (9.7)	13.6 (10.7)	0.80
Lifting and carrying					0.01
Almost all the time	75 (13.3%)	8 (10.8%)	15 (11.1%)	52 (14.6%)	
3/4 of the time	53 (9.4%)	1 (1.4%)	9 (6.7%)	43 (12.0%)	
1/2 of the time	102 (18.0%)	13 (17.6%)	22 (16.3%)	67 (18.8%)	
1/4 of the time	151 (26.7%)	16 (21.6%)	42 (31.1%)	93 (26.1%)	
Rarely/very little	157 (27.7%)	32 (43.2%)	37 (27.4%)	88 (24.6%)	
Never	28 (4.9%)	4 (5.4%)	10 (7.4%)	14 (3.9%)	
Highest pain intensity, 0–10	5.5 (2.9)	1.0 (1.2)	4.2 (2.1)	6.9 (2.1)	< 0.01
Number of pain sites	2.0 (1.2)	0.0 (0.0)	1.0 (0.0)	2.8 (0.8)	< 0.01

*DPhacto* Danish Physical activity cohort with objective measurements, *MVPA* Moderate to vigorous physical activity

Table 2 shows the mean and interquartile range for the HRV indices during work, leisure and sleep time for each group. The between groups comparison showed similar values for all indices during work, leisure and sleep time.

**Table 2:** Heart rate variability indices during work, leisure and sleep time for each group among the blue-collar participants in DPhacto. Data are presented as mean (interquartile range - IQR)

Variables	Work			Leisure			Sleep		
	Pain-free (*n* = 74)	Single-site pain (*n* = 136)	Multisite pain (*n* = 357)	Pain-free (*n* = 74)	Single-site pain (*n* = 136)	Multisite pain (*n* = 358)	Pain-free (*n* = 72)	Single-site pain (*n* = 135)	Multisite pain (*n* = 354)
IBI, ms	793.8 (136)	786.1 (130)	781.3 (136)	820.6 (132)	824.0 (133)	820.6 (132)	1068.3 (211)	1079.5 (212)	1075.7 (173)
SDNN, ms	53.5 (26)	54.7 (22)	54.7 (23)	52.4 (23)	53.0 (23)	53.6 (22)	54.2 (34)	52.9 (27)	56.8 (31)
ln SDNN	3.92 (0.51)	3.95 (0.40)	3.95 (0.43)	3.90 (0.46)	3.92 (0.45)	3.93 (0.42)	3.89 (0.66)	3.90 (0.52)	3.95 (0.58)
RMSSD, ms	26.9 (16)	26.2 (14)	26.6 (14)	27.6 (16)	27.3 (17)	28.5 (15)	47.5 (32)	47.0 (32)	51.7 (36)
ln RMSSD	3.17 (0.69)	3.17 (0.58)	3.17 (0.58)	3.20 (0.66)	3.21 (0.65)	3.25 (0.59)	3.69 (0.78)	3.71 (0.73)	3.79 (0.78)
LF, ms^2^/Hz	872.1 (732)	861.8 (691)	883.8 (752)	746.8 (692)	730.8 (592)	737.5 (648)	926.3 (944)	822.0 (749)	991.9 (902)
ln LF	6.49 (1.06)	6.54 (0.92)	6.52 (1.02)	6.34 (1.12)	6.37 (0.98)	6.35 (1.05)	6.39 (1.48)	6.38 (1.20)	6.46 (1.30)
HF, ms^2^/Hz	231.8 (213)	218.4 (168)	232.5 (195)	261.9 (214)	260.1 (244)	278.8 (248)	933.9 (865)	870.9 (942)	1133.8 (1094)
ln HF	4.94 (1.42)	4.92 (1.20)	4.97 (1.25)	5.08 (1.27)	5.11 (1.36)	5.19 (1.22)	6.18 (1.50)	6.24 (1.51)	6.37 (1.66)
LF/HF	6.08 (4)	6.70 (4)	6.36 (4)	4.97 (3)	5.31 (3)	4.97 (3)	1.97 (2)	1.75 (1)	1.90 (2)
ln LF/HF	1.66 (0.68)	1.75 (0.69)	1.69 (0.80)	1.47 (0.72)	1.52 (0.76)	1.43 (0.81)	0.32 (1.05)	0.20 (1.01)	0.17 (1.29)

*IBI* Interbeat intervals, *SDNN* Standard deviation of RR intervals, *ln* Natural logarithm, *RMSSD* Square root of the mean squared differences of successive RR intervals, *LF* Low-frequency power, *HF* High-frequency power, *LF/HF* Sympathovagal balance

The results from the linear mixed models showed no significant differences between groups and no significant interaction between groups and domains in the crude or adjusted models for any HRV index (Table 3). Significant differences between domains were found in the crude and adjusted model for all indices, except SDNN; sleep time showed higher values than leisure and work time, except for LF and LF/HF, which were higher during work.

**Table 3:** Estimates, standard error and *P* value from the linear mixed models for heart rate variability indices showing the main effect of group, domain and the interaction (group × domain) in the crude and adjusted models in DPhacto (*n* = 568)

Variables	Crude model			Adjusted model*		
	Estimate	Standard error	*P*	Estimate	Standard error	*P*
IBI, ms						
Group			0.93			0.58
Pain-free	793.8	13.5		935.3	43.4	
Single-site pain	786.1	9.9		936.6	42.4	
Multisite pain	781.1	6.1		930.6	42.0	
Domain			< 0.01			< 0.01
Sleep	294.7	4.4		293.1	4.5	
Leisure	39.5	4.4		37.9	4.5	
Interaction			0.36			0.28
Pain-free at sleep	−21.8	10.7		−21.6	11.0	
Pain-free at leisure	−12.6	10.6		−11.4	10.9	
Single-site pain at sleep	−1.2	8.4		5.5	8.8	
Single-site pain at leisure	−1.5	8.3		1.2	8.7	
ln SDNN						
Group			0.55			0.61
Pain-free	3.92	0.03		4.92	0.11	
Single-site pain	3.95	0.02		4.95	0.11	
Multisite pain	3.95	0.01		4.94	0.11	
Domain			0.20			0.07
Sleep	0.00	0.01		0.00	0.01	
Leisure	−0.01	0.01		−0.01	0.01	
Interaction			0.36			0.13
Pain-free at sleep	−0.04	0.03		−0.05	0.03	
Pain-free at leisure	0.00	0.03		0.00	0.03	
Single-site pain at sleep	−0.05	0.03		−0.07	0.03	
Single-site pain at leisure	−0.01	0.03		−0.01	0.03	
ln RMSSD						
Group			0.54			0.81
Pain-free	3.17	0.05		4.32	0.17	
Single-site pain	3.17	0.04		4.31	0.16	
Multisite pain	3.17	0.02		4.30	0.16	
Domain			< 0.01			< 0.01
Sleep	0.61	0.01		0.61	0.02	
Leisure	0.07	0.01		0.07	0.02	
Interaction			0.16			0.13
Pain-free at sleep	−0.10	0.04		− 0.11	0.04	
Pain-free at leisure	−0.04	0.04		−0.04	0.04	
Single-site pain at sleep	−0.06	0.03		−0.07	0.03	
Single-site pain at leisure	−0.03	0.03		−0.02	0.03	
ln LF						
Group			0.89			0.86
Pain-free	6.49	0.09		8.96	0.25	
Single-site pain	6.54	0.06		8.99	0.24	
Multisite pain	6.52	0.04		8.98	0.24	
Domain			< 0.01			< 0.01
Sleep	−0.06	0.03		−0.06	0.03	
Leisure	−0.17	0.03		−0.17	0.03	
Interaction			0.64			0.41
Pain-free at sleep	−0.04	0.09		− 0.07	0.09	
Pain-free at leisure	0.01	0.09		0.01	0.09	
Single-site pain at sleep	−0.09	0.07		−0.12	0.07	
Single-site pain at leisure	0.00	0.07		0.00	0.07	
ln HF						
Group			0.50			0.79
Pain-free	4.94	0.12		7.47	0.35	
Single-site pain	4.92	0.08		7.39	0.34	
Multisite pain	4.96	0.05		7.41	0.34	
Domain			< 0.01			< 0.01
Sleep	1.40	0.04		1.39	0.04	
Leisure	0.22	0.04		0.22	0.04	
Interaction			0.53			0.37
Pain-free at sleep	−0.17	0.10		−0.20	0.10	
Pain-free at leisure	−0.08	0.10		−0.07	0.10	
Single-site pain at sleep	−0.07	0.08		−0.08	0.08	
Single-site pain at leisure	−0.03	0.07		−0.02	0.08	
ln LF/HF						
Group			0.51			0.82
Pain-free	1.66	0.08		1.46	0.22	
Single-site pain	1.75	0.06		1.60	0.22	
Multisite pain	1.69	0.03		1.57	0.21	
Domain			< 0.01			< 0.01
Sleep	−1.51	0.03		−1.51	0.03	
Leisure	−0.26	0.03		−0.26	0.03	
Interaction			0.22			0.18
Pain-free at sleep	0.17	0.08		0.18	0.08	
Pain-free at leisure	0.07	0.08		0.06	0.08	
Single-site pain at sleep	−0.02	0.06		−0.04	0.07	
Single-site pain at leisure	0.03	0.06		0.02	0.07	

*IBI* Interbeat intervals, *SDNN* Standard deviation of RR intervals, *ln* Natural logarithm, *RMSSD* Square root of the mean squared differences of successive RR intervals, *LF* Low-frequency power, *HF* High-frequency power, *LF/HF* Sympathovagal balance. *Adjusted for: sex, age, BMI, smoking, moderate to vigorous physical activity at work and leisure, sitting time at work and leisure. Work domain was regarded as the reference

The sensitivity analysis, excluding workers with prescribed medication in the last three months (*n* = 234), showed no significant differences between groups and significant differences among domains for all indices in the crude and adjusted model, except for SDNN. The interaction between groups and domains was not significant for all indices. When using a more restrictive definition of a pain-free group, i.e., including in the pain-free group workers reporting no pain in all body parts (cut-off point = 0), the sensitivity analysis also showed no significant differences between groups, significant differences between domains (except for SDNN) and no significant interaction between groups and domains for all indices (Additional file 1: Table S1). The same results were found in the stratified analyses for age (Additional file 1: Table S2) and sex (Additional file 1: Table S3).

## Discussion

This cross-sectional study showed no association between multisite musculoskeletal pain and cardiac autonomic modulation during work, leisure and sleep.

Previous systematic reviews indicated that chronic pain is associated with a decrease in parasympathetic modulation [20, 40]. However, in agreement with our findings, Generaal et al. [14, 15] showed that autonomic cardiac modulation was not impaired with chronic multisite pain. One possible explanation for this divergence between the systematic reviews and the MSP studies could be that the results from the systematic reviews showing an autonomic imbalance in chronic pain are mainly based on studies with fibromyalgia patients. So, the autonomic imbalance involved in the pathophysiology of fibromyalgia [27] does not necessarily extend to active workers with multisite pain.

Another possible explanation for our findings can be related to the study population and data collection conditions. Most studies are based on patient samples evaluated in artificial conditions which can differ from active workers concerning physical, cognitive and psychosocial characteristics. Thus, it is possible that people with pain who is still at work are healthier, more physically active and have better coping mechanisms with their pain compared to clinical samples with pain [9, 24], which may be reflected in better autonomic function throughout daily living conditions, including work, leisure and sleep time. It is still possible that there are sub-groups of people with other features of multisite pain not captured in our study. Likewise, other factors like sleep disturbance, depressed mood, somatising tendency and psychosocial aspects of work may be involved in chronic multisite pain [6, 38]. These factors were not examined in our study, which is a limitation. Our findings may also suggest publication bias of positive results in previous studies [39].

The sensitivity analysis for medication yielded the same findings as the main analysis, including workers with prescribed medication. Additionally, the adoption of a more restrictive definition of pain-free resulted in the same findings. Also, stratified analysis on sex and age did not show association between multisite musculoskeletal pain and cardiac autonomic modulation during work, leisure and sleep.

The prevalence of multisite pain was very high in our sample, as it affected 63% of the DPhacto blue-collar workers, with the mean peak pain intensity of 6.9 points on a 0 to 10 scale. This high prevalence may be specific to the study population consisting of blue-collar workers, i.e., it refers to a disadvantaged socioeconomic group. Other studies have also shown a high prevalence of multisite pain in different populations, ranging from 35 to 64% [17, 30]. Although our findings showed that the imbalance of the autonomic modulation was not associated with multisite pain, this issue still deserves attention, since the theoretical framework support this relationship [25, 42]. Future studies should have a longitudinal design to verify if the autonomic imbalance precedes the occurrence of multisite pain.

### Strengths and limitations

A strength of our study is the large sample size, which allowed for stratified analyses. A further strength was the relatively homogenous group of blue-collar workers, which minimized potential socioeconomic confounding. The control for lifestyle factors such as smoking, physical activity, sitting time, and individual characteristics such as sex, age, and BMI is also highly relevant as these factors are closely related to HRV and pain. A potential limitation is the lack of control for the respiration rate, circadian variation, sleep quality and psychosocial aspects of work. Additionally, we have only looked at HRV during sleep, work and leisure, while there exist several other ways of evaluating autonomic function, such as assessing autonomic reactivity to functional tests (e.g. Valsalva, cold pressor and handgrip tests). Finally, the cross-sectional design of this study does not allow determining whether an autonomic imbalance may occur before the development of multisite pain.

## Conclusion

This cross-sectional study showed that multisite musculoskeletal pain is not associated with imbalanced cardiac autonomic regulation during work, leisure and sleep time.

## Additional file

Additional file 1: **Table S1.** Estimates, standard error and *P* value from the linear mixed models for heart rate variability indices showing the main effect of group, domain and the interaction (group × domain) in the crude and adjusted models using a strict definition of pain-free workers in DPhacto (*n* = 568). **Table S2.** Stratified analysis for age (< 50 years; ≥50 years). Estimates, standard error and *P* values from the linear mixed models for heart rate variability indices showing the main effect of group, domain and the interaction (group × domain) in the crude and adjusted models in DPhacto. **Table S3.** Stratified analysis for sex (male; female). Estimates, standard error and *P* values from the linear mixed models for heart rate variability indices showing the main effect of group, domain and the interaction (group × domain) in the crude and adjusted models in DPhacto. **Figure S1.** Original distribution of the HRV indices during work. **Figure S2.** Original distribution of the HRV indices during leisure. **Figure S3.** Original distribution of the HRV indices during sleep. (DOCX 1424 kb)

## Abbreviations

ANOVA: Analysis of variance
BMI: Body mass index
DPhacto: Danish Physical activity cohort with objective measurements
HF: High frequency
HRV: Heart rate variability
IBI: Inter beat intervals
LF: Low frequency
LF/HF: Sympathovagal balance
ln: Natural logarithm
MD: Mean difference
MSP: Multisite pain
MVPA: Moderate and vigorous physical activity
REML: Restricted maximum likelihood
RMSSD: Root mean square of successive differences of R-R intervals
SDNN: Standard deviation of R-R intervals
SE: Standard error
SSP: Single-site pain

## References

[1] Acharya UR, Paul JK, Kannathal N, Lim CM, Suri JS (2006). Heart rate variability: a review. Med Biol Eng Comput.

[2] Bernardi L, Valle F, Coco M, Calciati A, Sleight P (1996). Physical activity influences heart rate variability and very-low-frequency components in Holter electrocardiograms. Cardiovasc Res.

[3] Brage S, Brage N, Franks PW, Ekelund U, Wareham NJ (2005). Reliability and validity of the combined heart rate and movement sensor Actiheart. Eur J Clin Nutr.

[4] Carnes D, Parsons S, Ashby D, Breen A, Foster NE, Pincus T, Vogel S, Underwood M (2007). Chronic musculoskeletal pain rarely presents in a single body site: results from a UK population study. Rheumatology.

[5] Chan H-L, Lin M-A, Chao P-K, Lin C-H (2007). Correlates of the shift in heart rate variability with postures and walking by time-frequency analysis. Comp Meth Program Biomed.

[6] Coggon D, Ntani G (2017). Trajectories of multisite musculoskeletal pain and implications for prevention. Occup Environ Med.

[7] Coggon D, Ntani G, Palmer KT, Felli VE, Harari R, Barrero LH, Felknor SA, Gimeno D, Cattrell A, Vargas-Prada S, Bonzini M, Solidaki E, Merisalu E, Habib RR, Sadeghian F, Masood Kadir M, Warnakulasuriya SS, Matsudaira K, Nyantumbu B, Sim MR, Harcombe H, Cox K, Marziale MH, Sarquis LM, Harari F, Freire R, Harari N, Monroy MV, Quintana LA, Rojas M, Salazar Vega EJ, Harris EC, Serra C, Martinez JM, Delclos G, Benavides FG, Carugno M, Ferrario MM, Pesatori AC, Chatzi L, Bitsios P, Kogevinas M, Oha K, Sirk T, Sadeghian A, Peiris-John RJ, Sathiakumar N, Wickremasinghe AR, Yoshimura N, Kelsall HL, Hoe VC, Urquhart DM, Derrett S, McBride D, Herbison P, Gray A (2013). Patterns of multisite pain and associations with risk factors. Pain.

[8] Croft P, Dunn KM, Von Korff M (2007). Chronic pain syndromes: you can't have one without another. Pain.

[9] de Vries HJ, Brouwer S, Groothoff JW, Geertzen JH, Reneman MF (2011). Staying at work with chronic nonspecific musculoskeletal pain: a qualitative study of workers’ experiences. BMC Musculoskelet Disord.

[10] Dinas PC, Koutedakis Y, Flouris AD (2013). Effects of active and passive tobacco cigarette smoking on heart rate variability. Int J Cardiol.

[11] Eckhoff C, Straume B, Kvernmo S (2017). Multisite musculoskeletal pain in adolescence as a predictor of medical and social welfare benefits in young adulthood: the Norwegian Arctic adolescent health cohort study. Eur J Pain.

[12] Fayaz A, Croft P, Langford RM, Donaldson LJ, Jones GT (2016). Prevalence of chronic pain in the UK: a systematic review and meta-analysis of population studies. BMJ Open.

[13] Fernandes RCP, Burdorf A (2016). Associations of multisite pain with healthcare utilization, sickness absence and restrictions at work. Int Arch Occup Environ Health.

[14] Generaal E, Vogelzangs N, Macfarlane GJ, Geenen R, Smit JH, de Geus EJ, Penninx BW, Dekker J (2016). Biological stress systems, adverse life events and the onset of chronic multisite musculoskeletal pain: a 6-year cohort study. Ann Rheum Dis.

[15] Generaal E, Vogelzangs N, Macfarlane GJ, Geenen R, Smit JH, de Geus EJ, Dekker J, Penninx BW (2017). Biological stress systems, adverse life events, and the improvement of chronic multisite musculoskeletal pain across a 6-year follow-up. J Pain.

[16] Hallman DM, Birk Jørgensen M, Holtermann A (2017). On the health paradox of occupational and leisure-time physical activity using objective measurements: effects on autonomic imbalance. PLoS One.

[17] Haukka E, Kaila-Kangas L, Ojajärvi A, Saastamoinen P, Holtermann A, Jørgensen MB, Karppinen J, Heliövaara M, Leino-Arjas P (2015). Multisite musculoskeletal pain predicts medically certified disability retirement among Finns. Eur J Pain.

[18] Jørgensen MB, Korshøj M, Lagersted-Olsen J, Villumsen M, Mortensen OS, Skotte J, Søgaard K, Madeleine P, Thomsen BL, Holtermann A (2013). Physical activities at work and risk of musculoskeletal pain and its consequences: protocol for a study with objective field measures among blue-collar workers. BMC Musculoskelet Disord.

[19] Kamaleri Y, Natvig B, Ihlebaek CM, Benth JS, Bruusgaard D (2008). Number of pain sites is associated with demographic, lifestyle, and health-related factors in the general population. Eur J Pain.

[20] Koenig J, Falvay D, Clamor A, Wagner J, Jarczok MN, Ellis RJ, Weber C, Thayer JF (2016). Pneumogastric (vagus) nerve activity indexed by heart rate variability in chronic pain patients compared to healthy controls: a systematic review and meta-analysis. Pain Physician.

[21] Koenig J, Jarczok MN, Warth M, Ellis RJ, Bach C, Hillecke TK, Thayer JF (2014). Body mass index is related to autonomic nervous system activity as measured by heart rate variability – a replication using short term measurements. J Nutr Health Aging.

[22] Kristiansen Jesper, Korshøj Mette, Skotte Jørgen H, Jespersen Tobias, Søgaard Karen, Mortensen Ole S, Holtermann Andreas (2011). Comparison of two systems for long-term heart rate variability monitoring in free-living conditions - a pilot study. BioMedical Engineering OnLine.

[23] Kuorinka I, Jonsson B, Kilbom Å, Vinterberg H, Biering-Sörensen F, Anderson G, Jörgensen K (1987). Standardised Nordic questionnaires for the analysis of musculoskeletal symptoms. Appl Ergon.

[24] Linton SJ, Buer N (1995). Working despite pain: factors associated with work attendance versus dysfunction. Int J Behav Med.

[25] Maletic V, Raison CL (2009). Neurobiology of depression, fibromyalgia and neuropathic pain. Front Biosci.

[26] Malik M, Bigger JT, Camm AJ, Kleiger RE, Malliani A, Moss AJ, Schwartz PJ (1996). Heart rate variability standards of measurement, physiological interpretation, and clinical use. Eur Heart J.

[27] Martínez-Lavín M (2001). Is fibromyalgia a generalized reflex sympathetic dystrophy?. Clin Exp Rheumatol.

[28] Michael S, Graham KS, Oam DGM (2017). Cardiac autonomic responses during exercise and postexercise recovery using heart rate variability and systolic time intervals - a review. Front Physiol.

[29] Molfino A, Fiorentini A, Tubani L, Martuscelli M, Rossi Fanelli F, Laviano A (2009). Body mass index is related to autonomic nervous system activity as measured by heart rate variability. Eur J Clin Nutr.

[30] Neupane S, Leino-Arjas P, Nygård CH, Oakman J, Virtanen P (2017). Developmental pathways of multisite musculoskeletal pain: what is the influence of physical and psychosocial working conditions?. Occup Environ Med.

[31] Neupane S, Virtanen P, Leino-Arjas P, Miranda H, Siukola A, Nygård CH (2013). Multi-site pain and working conditions as predictors of work ability in a 4-year follow-up among food industry employees. Eur J Pain.

[32] Pan F, Laslett L, Blizzard L, Cicuttini F, Winzenberg T, Ding C, Jones G (2017). Associations between fat mass and multisite pain: a five-year longitudinal study. Arthritis Care Res.

[33] Perini R, Veicsteinas A (2003). Heart rate variability and autonomic activity at rest and during exercise in various physiological conditions. Eur J Appl Physiol.

[34] Phillips CJ (2009). The cost and burden of chronic pain. Rev Pain.

[35] Sato Tatiana O., Hallman David M., Kristiansen Jesper, Skotte Jørgen H., Holtermann Andreas (2017). Different autonomic responses to occupational and leisure time physical activities among blue-collar workers. International Archives of Occupational and Environmental Health.

[36] Skotte J, Korshøj M, Kristiansen J, Hanisch C, Holtermann A (2014). Detection of physical activity types using triaxial accelerometers. J Phys Act Health.

[37] Skotte Jørgen H, Kristiansen Jesper (2014). Heart rate variability analysis using robust period detection. BioMedical Engineering OnLine.

[38] Solidaki E, Chatzi L, Bitsios P, Markatzi I, Plana E, Castro F, Palmer K, Coggon D, Kogevinas M (2010). Work-related and psychological determinants of multisite musculoskeletal pain. Scand J Work Environ Health.

[39] Tak LM, Riese H, de Bock GH, Manoharan A, Kok IC, Rosmalen JG (2009). As good as it gets? A meta-analysis and systematic review of methodological quality of heart rate variability studies in functional somatic disorders. Biol Psychol.

[40] Tracy LM, Ioannou L, Baker KS, Gibson SJ, Georgiou-Karistianis N, Giummarra MJ (2016). Meta-analytic evidence for decreased heart rate variability in chronic pain implicating parasympathetic nervous system dysregulation. Pain.

[41] Walker-Bone K, Reading I, Coggon D, Cooper C, Palmer KT (2004). The anatomical pattern and determinants of pain in the neck and upper limbs: an epidemiologic study. Pain.

[42] Woda A, Picard P, Dutheil F (2016). Dysfunctional stress responses in chronic pain. Psychoneuroendocrinology.

