# Supplementary material for: The association between multisite musculoskeletal pain and cardiac autonomic modulation during work, leisure and sleep – a cross-sectional study
Source: BMC Musculoskelet Disord. 2018 Nov 20;19:405. doi: 10.1186/s12891-018-2312-3 (PMC6247621; doi:10.1186/s12891-018-2312-3)
Supplement: Supplementary file 1 — Table S1. Estimates, standard error and P value from the linear mixed models for heart rate variability indices showing the main effect of group, domain and the interaction (group × domain) in the crude and adjusted models using a strict definition of pain-free workers in DPhacto (n = 568). Table S2. Stratified analysis for age (< 50 years; ≥50 years). Estimates, standard error and P values from the linear mixed models for heart rate variability indices showing the main effect of group, domain and the interaction (group × domain) in the crude and adjusted models in DPhacto. Table S3. Stratified analysis for sex (male; female). Estimates, standard error and P values from the linear mixed models for heart rate variability indices showing the main effect of group, domain and the interaction (group × domain) in the crude and adjusted models in DPhacto. Figure S1. Original distribution of the HRV indices during work. Figure S2. Original distribution of the HRV indices during leisure. Figure S3. Original distribution of the HRV indices during sleep. (DOCX 1424 kb) [file 12891_2018_2312_MOESM1_ESM.docx]

**Table S1** Estimates, standard error and *P* value from the linear mixed models for heart rate variability indices showing the main effect of group, domain and the interaction (group × domain) in the crude and adjusted models using a strict definition of pain-free workers in DPhacto (n=568).

| **Variables** |  | **Crude model** | | |  | **Adjusted model*** | | |
| --- | --- | --- | --- | --- | --- | --- | --- | --- |
|  |  | **Estimate** | **Standard error** | ***P*** |  | **Estimate** | **Standard error** | ***P*** |
| IBI, ms |  |  |  |  |  |  |  |  |
| Group |  |  |  | 0.93 |  |  |  | 0.73 |
| Pain-free |  | 788.2 | 18.3 |  |  | 930.5 | 45.4 |  |
| Single-site pain |  | 795.5 | 12.8 |  |  | 945.9 | 43.3 |  |
| Multisite pain |  | 781.4 | 5.5 |  |  | 931.1 | 41.8 |  |
| Domain |  |  |  | <0.01 |  |  |  | <0.01 |
| Sleep |  | 297.0 | 3.9 |  |  | 296.1 | 4.1 |  |
| Leisure |  | 41.3 | 3.9 |  |  | 40.2 | 4.0 |  |
| Interaction |  |  |  | 0.04 |  |  |  | 0.11 |
| Pain-free at sleep |  | -26.8 | 13.8 |  |  | -26.2 | 14.3 |  |
| Pain-free at leisure |  | -12.9 | 13.7 |  |  | -10.3 | 14.1 |  |
| Single-site pain at sleep |  | -24.1 | 10.0 |  |  | -19.7 | 10.4 |  |
| Single-site pain at leisure |  | -20.7 | 9.9 |  |  | -19.0 | 10.4 |  |
| ln SDNN |  |  |  |  |  |  |  |  |
| Group |  |  |  | 0.58 |  |  |  | 0.87 |
| Pain-free |  | 3.90 | 0.05 |  |  | 4.91 | 0.12 |  |
| Single-site pain |  | 3.95 | 0.03 |  |  | 4.95 | 0.11 |  |
| Multisite pain |  | 3.95 | 0.01 |  |  | 4.93 | 0.10 |  |
| Domain |  |  |  | 0.34 |  |  |  | 0.29 |
| Sleep |  | 0.00 | 0.01 |  |  | 0.00 | 0.01 |  |
| Leisure |  | -0.01 | 0.01 |  |  | -0.01 | 0.01 |  |
| Interaction |  |  |  | 0.65 |  |  |  | 0.60 |
| Pain-free at sleep |  | 0.00 | 0.04 |  |  | 0.00 | 0.05 |  |
| Pain-free at leisure |  | 0.00 | 0.04 |  |  | 0.00 | 0.05 |  |
| Single-site pain at sleep |  | -0.05 | 0.03 |  |  | -0.06 | 0.03 |  |
| Single-site pain at leisure |  | -0.02 | 0.03 |  |  | -0.02 | 0.03 |  |
| ln RMSSD |  |  |  |  |  |  |  |  |
| Group |  |  |  | 0.44 |  |  |  | 0.78 |
| Pain-free |  | 3.11 | 0.07 |  |  | 4.27 | 0.18 |  |
| Single-site pain |  | 3.19 | 0.05 |  |  | 4.33 | 0.17 |  |
| Multisite pain |  | 3.17 | 0.02 |  |  | 4.29 | 0.16 |  |
| Domain |  |  |  | <0.01 |  |  |  | <0.01 |
| Sleep |  | 0.59 | 0.01 |  |  | 0.59 | 0.01 |  |
| Leisure |  | 0.07 | 0.01 |  |  | 0.07 | 0.01 |  |
| Interaction |  |  |  | 0.54 |  |  |  | 0.49 |
| Pain-free at sleep |  | -0.04 | 0.06 |  |  | -0.05 | 0.06 |  |
| Pain-free at leisure |  | -0.02 | 0.06 |  |  | -0.02 | 0.06 |  |
| Single-site pain at sleep |  | -0.06 | 0.04 |  |  | -0.07 | 0.04 |  |
| Single-site pain at leisure |  | -0.06 | 0.04 |  |  | -0.06 | 0.04 |  |
| ln LF |  |  |  |  |  |  |  |  |
| Group |  |  |  | 0.89 |  |  |  | 0.99 |
| Pain-free |  | 6.44 | 0.12 |  |  | 8.94 | 0.26 |  |
| Single-site pain |  | 6.56 | 0.08 |  |  | 9.02 | 0.25 |  |
| Multisite pain |  | 6.52 | 0.03 |  |  | 8.97 | 0.24 |  |
| Domain |  |  |  | <0.01 |  |  |  | <0.01 |
| Sleep |  | -0.08 | 0.03 |  |  | -0.09 | 0.03 |  |
| Leisure |  | -0.15 | 0.03 |  |  | -0.16 | 0.03 |  |
| Interaction |  |  |  | 0.81 |  |  |  | 0.85 |
| Pain-free at sleep |  | 0.06 | 0.11 |  |  | 0.05 | 0.12 |  |
| Pain-free at leisure |  | 0.01 | 0.11 |  |  | 0.01 | 0.12 |  |
| Single-site pain at sleep |  | -0.09 | 0.08 |  |  | -0.09 | 0.09 |  |
| Single-site pain at leisure |  | -0.05 | 0.08 |  |  | -0.04 | 0.09 |  |
| ln HF |  |  |  |  |  |  |  |  |
| Group |  |  |  | 0.44 |  |  |  | 0.83 |
| Pain-free |  | 4.83 | 0.16 |  |  | 7.38 | 0.37 |  |
| Single-site pain |  | 4.97 | 0.11 |  |  | 7.46 | 0.35 |  |
| Multisite pain |  | 4.96 | 0.04 |  |  | 7.39 | 0.34 |  |
| Domain |  |  |  | <0.01 |  |  |  | <0.01 |
| Sleep |  | 1.38 | 0.03 |  |  | 1.37 | 0.03 |  |
| Leisure |  | 0.23 | 0.03 |  |  | 0.22 | 0.03 |  |
| Interaction |  |  |  | 0.56 |  |  |  | 0.49 |
| Pain-free at sleep |  | -0.14 | 0.13 |  |  | -0.17 | 0.13 |  |
| Pain-free at leisure |  | -0.03 | 0.13 |  |  | -0.02 | 0.13 |  |
| Single-site pain at sleep |  | -0.09 | 0.09 |  |  | -0.10 | 0.09 |  |
| Single-site pain at leisure |  | -0.12 | 0.09 |  |  | -0.11 | 0.09 |  |
| ln LF/HF |  |  |  |  |  |  |  |  |
| Group |  |  |  | 0.43 |  |  |  | 0.75 |
| Pain-free |  | 1.73 | 0.11 |  |  | 1.53 | 0.24 |  |
| Single-site pain |  | 1.70 | 0.07 |  |  | 1.53 | 0.22 |  |
| Multisite pain |  | 1.70 | 0.03 |  |  | 1.57 | 0.21 |  |
| Domain |  |  |  | <0.01 |  |  |  | <0.01 |
| Sleep |  | -1.52 | 0.03 |  |  | -1.52 | 0.03 |  |
| Leisure |  | -0.26 | 0.03 |  |  | -0.26 | 0.03 |  |
| Interaction |  |  |  | 0.08 |  |  |  | 0.04 |
| Pain-free at sleep |  | 0.26 | 0.11 |  |  | 0.29 | 0.11 |  |
| Pain-free at leisure |  | 0.02 | 0.11 |  |  | 0.01 | 0.11 |  |
| Single-site pain at sleep |  | 0.00 | 0.08 |  |  | 0.02 | 0.08 |  |
| Single-site pain at leisure |  | 0.07 | 0.08 |  |  | 0.08 | 0.08 |  |

IBI: interbeat intervals; SDNN: standard deviation of RR intervals; ln: natural logarithm; RMSSD: square root of the mean squared differences of successive RR intervals; LF: low-frequency power; HF: high-frequency power; LF/HF: sympathovagal balance. *Adjusted for: sex, age, BMI, smoking, moderate to vigorous physical activity at work and leisure, sitting time at work and leisure. Work domain was regarded as the reference.

**Table S2.** Stratified analysis for age (<50 years; ≥50 years). Estimates, standard error and *P* values from the linear mixed models for heart rate variability indices showing the main effect of group, domain and the interaction (group × domain) in the crude and adjusted models in DPhacto.

| **Variables** |  | **Age < 50 years (n=353)** | | | | | | |  | **Age ≥ 50 years (n=215)** | | | | | | |
| --- | --- | --- | --- | --- | --- | --- | --- | --- | --- | --- | --- | --- | --- | --- | --- | --- |
|  |  | **Crude model** | | |  | **Adjusted model*** | | |  | **Crude model** | | |  | **Adjusted model*** | | |
|  |  | **Estimate** | **Standard error** | ***P*** |  | **Estimate** | **Standard error** | ***P*** |  | **Estimate** | **Standard error** | ***P*** |  | **Estimate** | **Standard error** | ***P*** |
| IBI, ms |  |  |  |  |  |  |  |  |  |  |  |  |  |  |  |  |
| Group |  |  |  | 0.34 |  |  |  | 0.19 |  |  |  | 0.40 |  |  |  | 0.84 |
| Pain-free |  | 767.7 | 17.4 |  |  | 960.8 | 52.9 |  |  | 830.0 | 21.0 |  |  | 700.9 | 141.6 |  |
| Single-site pain |  | 781.4 | 11.8 |  |  | 978.7 | 51.2 |  |  | 796.1 | 17.8 |  |  | 681.3 | 140.3 |  |
| Multisite pain |  | 776.1 | 7.7 |  |  | 977.1 | 50.7 |  |  | 788.8 | 9.8 |  |  | 673.1 | 137.9 |  |
| Domain |  |  |  | <0.01 |  |  |  | <0.01 |  |  |  | <0.01 |  |  |  | <0.01 |
| Sleep |  | 300.1 | 5.7 |  |  | 299.9 | 5.8 |  |  | 286.4 | 6.7 |  |  | 282.1 | 7.0 |  |
| Leisure |  | 34.9 | 5.7 |  |  | 33.4 | 5.8 |  |  | 46.5 | 6.6 |  |  | 45.2 | 6.9 |  |
| Interaction |  |  |  | 0.45 |  |  |  | 0.25 |  |  |  | 0.06 |  |  |  | 0.09 |
| Pain-free at sleep |  | -12.8 | 14.1 |  |  | -13.0 | 14.3 |  |  | -33.4 | 15.8 |  |  | -33.3 | 16.5 |  |
| Pain-free at leisure |  | -9.8 | 14.0 |  |  | -7.8 | 14.2 |  |  | -17.3 | 15.6 |  |  | -17.2 | 16.3 |  |
| Single-site pain at sleep |  | 14.5 | 10.4 |  |  | 19.5 | 10.7 |  |  | -37.9 | 13.7 |  |  | -31.8 | 14.8 |  |
| Single-site pain at leisure |  | 4.4 | 10.4 |  |  | 5.1 | 10.7 |  |  | -11.7 | 13.7 |  |  | -4.2 | 14.8 |  |
| ln SDNN |  |  |  |  |  |  |  |  |  |  |  |  |  |  |  |  |
| Group |  |  |  | 0.13 |  |  |  | 0.07 |  |  |  | 0.53 |  |  |  | 0.48 |
| Pain-free |  | 3.93 | 0.05 |  |  | 4.80 | 0.14 |  |  | 3.91 | 0.05 |  |  | 4.99 | 0.36 |  |
| Single-site pain |  | 4.02 | 0.03 |  |  | 4.91 | 0.13 |  |  | 3.82 | 0.05 |  |  | 4.90 | 0.36 |  |
| Multisite pain |  | 4.02 | 0.02 |  |  | 4.90 | 0.13 |  |  | 3.83 | 0.02 |  |  | 4.89 | 0.35 |  |
| Domain |  |  |  | 0.75 |  |  |  | 0.58 |  |  |  | 0.16 |  |  |  | 0.06 |
| Sleep |  | 0.01 | 0.02 |  |  | 0.01 | 0.02 |  |  | -0.01 | 0.02 |  |  | 0.00 | 0.02 |  |
| Leisure |  | -0.02 | 0.02 |  |  | -0.02 | 0.02 |  |  | 0.00 | 0.02 |  |  | 0.00 | 0.02 |  |
| Interaction |  |  |  | 0.27 |  |  |  | 0.21 |  |  |  | 0.74 |  |  |  | 0.45 |
| Pain-free at sleep |  | -0.01 | 0.05 |  |  | -0.02 | 0.05 |  |  | -0.07 | 0.05 |  |  | -0.10 | 0.06 |  |
| Pain-free at leisure |  | 0.01 | 0.05 |  |  | 0.02 | 0.05 |  |  | -0.03 | 0.05 |  |  | -0.03 | 0.06 |  |
| Single-site pain at sleep |  | -0.07 | 0.03 |  |  | -0.07 | 0.03 |  |  | -0.02 | 0.05 |  |  | -0.06 | 0.05 |  |
| Single-site pain at leisure |  | 0.00 | 0.03 |  |  | 0.00 | 0.03 |  |  | -0.03 | 0.05 |  |  | -0.04 | 0.05 |  |
| ln RMSSD |  |  |  |  |  |  |  |  |  |  |  |  |  |  |  |  |
| Group |  |  |  | 0.20 |  |  |  | 0.30 |  |  |  | 0.37 |  |  |  | 0.47 |
| Pain-free |  | 3.17 | 0.07 |  |  | 4.23 | 0.22 |  |  | 3.18 | 0.08 |  |  | 4.14 | 0.51 |  |
| Single-site pain |  | 3.24 | 0.04 |  |  | 4.32 | 0.21 |  |  | 3.01 | 0.06 |  |  | 3.99 | 0.51 |  |
| Multisite pain |  | 3.27 | 0.03 |  |  | 4.31 | 0.21 |  |  | 3.02 | 0.03 |  |  | 3.98 | 0.50 |  |
| Domain |  |  |  | <0.01 |  |  |  | <0.01 |  |  |  | <0.01 |  |  |  | <0.01 |
| Sleep |  | 0.65 | 0.02 |  |  | 0.64 | 0.02 |  |  | 0.55 | 0.03 |  |  | 0.56 | 0.03 |  |
| Leisure |  | 0.07 | 0.02 |  |  | 0.06 | 0.02 |  |  | 0.08 | 0.03 |  |  | 0.09 | 0.03 |  |
| Interaction |  |  |  | 0.65 |  |  |  | 0.56 |  |  |  | 0.12 |  |  |  | 0.15 |
| Pain-free at sleep |  | -0.04 | 0.06 |  |  | -0.05 | 0.06 |  |  | -0.18 | 0.07 |  |  | -0.18 | 0.07 |  |
| Pain-free at leisure |  | -0.01 | 0.06 |  |  | -0.01 | 0.06 |  |  | -0.09 | 0.07 |  |  | -0.08 | 0.07 |  |
| Single-site pain at sleep |  | -0.06 | 0.04 |  |  | -0.07 | 0.04 |  |  | -0.08 | 0.06 |  |  | -0.10 | 0.07 |  |
| Single-site pain at leisure |  | 0.00 | 0.04 |  |  | 0.00 | 0.04 |  |  | -0.08 | 0.06 |  |  | -0.06 | 0.07 |  |
| ln LF |  |  |  |  |  |  |  |  |  |  |  |  |  |  |  |  |
| Group |  |  |  | 0.46 |  |  |  | 0.21 |  |  |  | 0.36 |  |  |  | 0.21 |
| Pain-free |  | 6.55 | 0.11 |  |  | 8.62 | 0.30 |  |  | 6.40 | 0.14 |  |  | 9.76 | 0.86 |  |
| Single-site pain |  | 6.71 | 0.07 |  |  | 8.82 | 0.29 |  |  | 6.15 | 0.11 |  |  | 9.48 | 0.86 |  |
| Multisite pain |  | 6.71 | 0.05 |  |  | 8.79 | 0.28 |  |  | 6.23 | 0.06 |  |  | 9.53 | 0.84 |  |
| Domain |  |  |  | <0.01 |  |  |  | <0.01 |  |  |  | <0.01 |  |  |  | <0.01 |
| Sleep |  | -0.10 | 0.04 |  |  | -0.11 | 0.05 |  |  | 0.00 | 0.05 |  |  | 0.00 | 0.06 |  |
| Leisure |  | -0.15 | 0.04 |  |  | -0.16 | 0.05 |  |  | -0.18 | 0.05 |  |  | -0.18 | 0.05 |  |
| Interaction |  |  |  | 0.77 |  |  |  | 0.71 |  |  |  | 0.90 |  |  |  | 0.69 |
| Pain-free at sleep |  | 0.00 | 0.12 |  |  | -0.02 | 0.12 |  |  | -0.11 | 0.13 |  |  | -0.14 | 0.14 |  |
| Pain-free at leisure |  | 0.07 | 0.12 |  |  | 0.07 | 0.12 |  |  | -0.06 | 0.13 |  |  | -0.06 | 0.14 |  |
| Single-site pain at sleep |  | -0.08 | 0.09 |  |  | -0.09 | 0.09 |  |  | -0.08 | 0.11 |  |  | -0.16 | 0.12 |  |
| Single-site pain at leisure |  | 0.01 | 0.09 |  |  | 0.02 | 0.09 |  |  | -0.02 | 0.11 |  |  | -0.05 | 0.12 |  |
| ln HF |  |  |  |  |  |  |  |  |  |  |  |  |  |  |  |  |
| Group |  |  |  | 0.16 |  |  |  | 0.34 |  |  |  | 0.58 |  |  |  | 0.29 |
| Pain-free |  | 4.99 | 0.15 |  |  | 7.24 | 0.46 |  |  | 4.87 | 0.17 |  |  | 7.28 | 1.05 |  |
| Single-site pain |  | 5.08 | 0.10 |  |  | 7.39 | 0.44 |  |  | 4.57 | 0.14 |  |  | 6.87 | 1.04 |  |
| Multisite pain |  | 5.21 | 0.06 |  |  | 7.43 | 0.44 |  |  | 4.59 | 0.08 |  |  | 6.84 | 1.02 |  |
| Domain |  |  |  | <0.01 |  |  |  | <0.01 |  |  |  | <0.01 |  |  |  | <0.01 |
| Sleep |  | 1.45 | 0.05 |  |  | 1.44 | 0.05 |  |  | 1.32 | 0.06 |  |  | 1.32 | 0.06 |  |
| Leisure |  | 0.22 | 0.05 |  |  | 0.20 | 0.05 |  |  | 0.24 | 0.06 |  |  | 0.24 | 0.06 |  |
| Interaction |  |  |  | 0.95 |  |  |  | 0.92 |  |  |  | 0.19 |  |  |  | 0.11 |
| Pain-free at sleep |  | -0.02 | 0.13 |  |  | -0.03 | 0.13 |  |  | -0.37 | 0.15 |  |  | -0.44 | 0.16 |  |
| Pain-free at leisure |  | -0.03 | 0.13 |  |  | -0.02 | 0.13 |  |  | -0.14 | 0.15 |  |  | -0.14 | 0.16 |  |
| Single-site pain at sleep |  | -0.06 | 0.09 |  |  | -0.08 | 0.10 |  |  | -0.11 | 0.13 |  |  | -0.13 | 0.14 |  |
| Single-site pain at leisure |  | 0.00 | 0.09 |  |  | 0.00 | 0.10 |  |  | -0.11 | 0.13 |  |  | -0.06 | 0.14 |  |
| ln LF/HF |  |  |  |  |  |  |  |  |  |  |  |  |  |  |  |  |
| Group |  |  |  | 0.23 |  |  |  | 0.63 |  |  |  | 0.99 |  |  |  | 0.54 |
| Pain-free |  | 1.70 | 0.10 |  |  | 1.36 | 0.29 |  |  | 1.62 | 0.12 |  |  | 2.33 | 0.67 |  |
| Single-site pain |  | 1.76 | 0.07 |  |  | 1.42 | 0.28 |  |  | 1.72 | 0.10 |  |  | 2.52 | 0.66 |  |
| Multisite pain |  | 1.63 | 0.04 |  |  | 1.35 | 0.27 |  |  | 1.78 | 0.05 |  |  | 2.60 | 0.65 |  |
| Domain |  |  |  | <0.01 |  |  |  | <0.01 |  |  |  | <0.01 |  |  |  | <0.01 |
| Sleep |  | -1.61 | 0.04 |  |  | -1.61 | 0.04 |  |  | -1.36 | 0.05 |  |  | -1.35 | 0.05 |  |
| Leisure |  | -0.23 | 0.04 |  |  | -0.22 | 0.04 |  |  | -0.31 | 0.05 |  |  | -0.32 | 0.05 |  |
| Interaction |  |  |  | 0.98 |  |  |  | 0.99 |  |  |  | 0.06 |  |  |  | 0.04 |
| Pain-free at sleep |  | 0.03 | 0.11 |  |  | 0.02 | 0.11 |  |  | 0.35 | 0.13 |  |  | 0.40 | 0.13 |  |
| Pain-free at leisure |  | 0.05 | 0.11 |  |  | 0.03 | 0.11 |  |  | 0.10 | 0.13 |  |  | 0.11 | 0.13 |  |
| Single-site pain at sleep |  | -0.03 | 0.08 |  |  | -0.02 | 0.08 |  |  | 0.04 | 0.11 |  |  | -0.01 | 0.12 |  |
| Single-site pain at leisure |  | 0.00 | 0.08 |  |  | 0.00 | 0.08 |  |  | 0.10 | 0.11 |  |  | 0.04 | 0.12 |  |

IBI: interbeat intervals; SDNN: standard deviation of RR intervals; ln: natural logarithm; RMSSD: square root of the mean squared differences of successive RR intervals; LF: low-frequency power; HF: high-frequency power; LF/HF: sympathovagal balance. *Adjusted for: sex, age, BMI, smoking, moderate to vigorous physical activity at work and leisure, sitting time at work and leisure. Work domain was regarded as the reference.

**Table S3.** Stratified analysis for sex (male; female). Estimates, standard error and *P* values from the linear mixed models for heart rate variability indices showing the main effect of group, domain and the interaction (group × domain) in the crude and adjusted models in DPhacto.

| **Variables** |  | **Male (n=321)** | | | | | | |  | **Female (n=247)** | | | | | | |
| --- | --- | --- | --- | --- | --- | --- | --- | --- | --- | --- | --- | --- | --- | --- | --- | --- |
|  |  | **Crude model** | | |  | **Adjusted model*** | | |  | **Crude model** | | |  | **Adjusted model*** | | |
|  |  | **Estimate** | **Standard error** | ***P*** |  | **Estimate** | **Standard error** | ***P*** |  | **Estimate** | **Standard error** | ***P*** |  | **Estimate** | **Standard error** | ***P*** |
| IBI, ms |  |  |  |  |  |  |  |  |  |  |  |  |  |  |  |  |
| Group |  |  |  | 0.33 |  |  |  | 0.19 |  |  |  | 0.45 |  |  |  | 0.62 |
| Pain-free |  | 794.1 | 16.6 |  |  | 882.6 | 56.6 |  |  | 793.2 | 22.1 |  |  | 859.0 | 66.6 |  |
| Single-site pain |  | 805.6 | 13.0 |  |  | 898.4 | 54.4 |  |  | 759.0 | 14.6 |  |  | 837.6 | 65.4 |  |
| Multisite pain |  | 789.9 | 8.3 |  |  | 875.3 | 53.5 |  |  | 770.8 | 8.6 |  |  | 853.5 | 64.1 |  |
| Domain |  |  |  | <0.01 |  |  |  | <0.01 |  |  |  | <0.01 |  |  |  | <0.01 |
| Sleep |  | 316.2 | 6.2 |  |  | 313.2 | 6.4 |  |  | 269.7 | 5.8 |  |  | 269.5 | 6.0 |  |
| Leisure |  | 38.1 | 6.2 |  |  | 35.8 | 6.4 |  |  | 41.1 | 5.8 |  |  | 40.5 | 6.0 |  |
| Interaction |  |  |  | 0.19 |  |  |  | 0.16 |  |  |  | 0.79 |  |  |  | 0.86 |
| Pain-free at sleep |  | -32.5 | 13.9 |  |  | -31.9 | 14.2 |  |  | -17.0 | 16.0 |  |  | -17.9 | 16.9 |  |
| Pain-free at leisure |  | -10.1 | 13.7 |  |  | -9.2 | 14.0 |  |  | -16.6 | 16.0 |  |  | -14.1 | 16.8 |  |
| Single-site pain at sleep |  | 0.28 | 11.5 |  |  | 6.2 | 12.0 |  |  | -7.4 | 11.5 |  |  | -0.2 | 12.2 |  |
| Single-site pain at leisure |  | 1.80 | 11.4 |  |  | 2.8 | 11.9 |  |  | -5.9 | 11.5 |  |  | -0.4 | 12.2 |  |
| ln SDNN |  |  |  |  |  |  |  |  |  |  |  |  |  |  |  |  |
| Group |  |  |  | 0.35 |  |  |  | 0.55 |  |  |  | 0.31 |  |  |  | 0.53 |
| Pain-free |  | 3.93 | 0.04 |  |  | 4.77 | 0.14 |  |  | 3.90 | 0.06 |  |  | 4.78 | 0.18 |  |
| Single-site pain |  | 4.03 | 0.03 |  |  | 4.84 | 0.14 |  |  | 3.85 | 0.04 |  |  | 4.77 | 0.17 |  |
| Multisite pain |  | 3.99 | 0.02 |  |  | 4.80 | 0.13 |  |  | 3.90 | 0.02 |  |  | 4.79 | 0.17 |  |
| Domain |  |  |  | 0.04 |  |  |  | <0.01 |  |  |  | 0.38 |  |  |  | 0.56 |
| Sleep |  | -0.02 | 0.02 |  |  | -0.02 | 0.02 |  |  | 0.04 | 0.02 |  |  | 0.03 | 0.02 |  |
| Leisure |  | -0.02 | 0.02 |  |  | -0.02 | 0.02 |  |  | 0.00 | 0.02 |  |  | -0.01 | 0.02 |  |
| Interaction |  |  |  | 0.68 |  |  |  | 0.38 |  |  |  | 0.74 |  |  |  | 0.65 |
| Pain-free at sleep |  | -0.02 | 0.04 |  |  | -0.04 | 0.04 |  |  | -0.04 | 0.06 |  |  | -0.05 | 0.06 |  |
| Pain-free at leisure |  | 0.01 | 0.04 |  |  | 0.01 | 0.04 |  |  | -0.03 | 0.06 |  |  | -0.03 | 0.06 |  |
| Single-site pain at sleep |  | -0.05 | 0.04 |  |  | -0.07 | 0.04 |  |  | -0.05 | 0.04 |  |  | -0.06 | 0.04 |  |
| Single-site pain at leisure |  | -0.01 | 0.04 |  |  | -0.02 | 0.04 |  |  | -0.01 | 0.04 |  |  | 0.00 | 0.04 |  |
| ln RMSSD |  |  |  |  |  |  |  |  |  |  |  |  |  |  |  |  |
| Group |  |  |  | 0.57 |  |  |  | 0.62 |  |  |  | 0.12 |  |  |  | 0.27 |
| Pain-free |  | 3.17 | 0.07 |  |  | 4.21 | 0.22 |  |  | 3.18 | 0.09 |  |  | 4.38 | 0.27 |  |
| Single-site pain |  | 3.22 | 0.05 |  |  | 4.26 | 0.21 |  |  | 3.09 | 0.06 |  |  | 4.32 | 0.26 |  |
| Multisite pain |  | 3.17 | 0.03 |  |  | 4.20 | 0.21 |  |  | 3.18 | 0.03 |  |  | 4.38 | 0.26 |  |
| Domain |  |  |  | <0.01 |  |  |  | <0.01 |  |  |  | <0.01 |  |  |  | <0.01 |
| Sleep |  | 0.56 | 0.02 |  |  | 0.56 | 0.02 |  |  | 0.67 | 0.02 |  |  | 0.67 | 0.02 |  |
| Leisure |  | 0.04 | 0.02 |  |  | 0.04 | 0.02 |  |  | 0.11 | 0.02 |  |  | 0.11 | 0.02 |  |
| Interaction |  |  |  | 0.50 |  |  |  | 0.54 |  |  |  | 0.34 |  |  |  | 0.45 |
| Pain-free at sleep |  | -0.11 | 0.06 |  |  | -0.10 | 0.06 |  |  | -0.06 | 0.07 |  |  | -0.08 | 0.07 |  |
| Pain-free at leisure |  | -0.02 | 0.06 |  |  | -0.01 | 0.06 |  |  | -0.07 | 0.07 |  |  | -0.06 | 0.07 |  |
| Single-site pain at sleep |  | -0.02 | 0.05 |  |  | -0.04 | 0.05 |  |  | -0.10 | 0.05 |  |  | -0.09 | 0.05 |  |
| Single-site pain at leisure |  | 0.00 | 0.05 |  |  | -0.01 | 0.05 |  |  | -0.05 | 0.05 |  |  | -0.04 | 0.05 |  |
| ln LF |  |  |  |  |  |  |  |  |  |  |  |  |  |  |  |  |
| Group |  |  |  | 0.51 |  |  |  | 0.84 |  |  |  | 0.58 |  |  |  | 0.88 |
| Pain-free |  | 6.53 | 0.11 |  |  | 8.63 | 0.33 |  |  | 6.41 | 0.15 |  |  | 8.38 | 0.39 |  |
| Single-site pain |  | 6.70 | 0.08 |  |  | 8.70 | 0.32 |  |  | 6.31 | 0.10 |  |  | 8.35 | 0.38 |  |
| Multisite pain |  | 6.61 | 0.05 |  |  | 8.65 | 0.31 |  |  | 6.42 | 0.06 |  |  | 8.39 | 0.37 |  |
| Domain |  |  |  | <0.01 |  |  |  | <0.01 |  |  |  | <0.01 |  |  |  | <0.01 |
| Sleep |  | -0.03 | 0.04 |  |  | -0.03 | 0.05 |  |  | -0.10 | 0.05 |  |  | -0.10 | 0.06 |  |
| Leisure |  | -0.13 | 0.04 |  |  | -0.14 | 0.05 |  |  | -0.20 | 0.05 |  |  | -0.21 | 0.06 |  |
| Interaction |  |  |  | 0.44 |  |  |  | 0.24 |  |  |  | 0.96 |  |  |  | 0.96 |
| Pain-free at sleep |  | -0.11 | 0.11 |  |  | -0.13 | 0.11 |  |  | 0.04 | 0.15 |  |  | 0.02 | 0.16 |  |
| Pain-free at leisure |  | 0.02 | 0.11 |  |  | 0.03 | 0.11 |  |  | -0.02 | 0.15 |  |  | -0.04 | 0.16 |  |
| Single-site pain at sleep |  | -0.14 | 0.09 |  |  | -0.18 | 0.09 |  |  | -0.02 | 0.11 |  |  | -0.04 | 0.12 |  |
| Single-site pain at leisure |  | -0.01 | 0.09 |  |  | -0.01 | 0.09 |  |  | 0.03 | 0.11 |  |  | 0.02 | 0.12 |  |
| ln HF |  |  |  |  |  |  |  |  |  |  |  |  |  |  |  |  |
| Group |  |  |  | 0.57 |  |  |  | 0.79 |  |  |  | 0.08 |  |  |  | 0.17 |
| Pain-free |  | 4.88 | 0.15 |  |  | 7.50 | 0.47 |  |  | 5.06 | 0.19 |  |  | 7.71 | 0.53 |  |
| Single-site pain |  | 4.96 | 0.12 |  |  | 7.52 | 0.45 |  |  | 4.85 | 0.12 |  |  | 7.55 | 0.52 |  |
| Multisite pain |  | 4.87 | 0.07 |  |  | 7.43 | 0.44 |  |  | 5.08 | 0.07 |  |  | 7.75 | 0.51 |  |
| Domain |  |  |  | <0.01 |  |  |  | <0.01 |  |  |  | <0.01 |  |  |  | <0.01 |
| Sleep |  | 1.33 | 0.06 |  |  | 1.33 | 0.06 |  |  | 1.47 | 0.05 |  |  | 1.47 | 0.06 |  |
| Leisure |  | 0.16 | 0.05 |  |  | 0.15 | 0.06 |  |  | 0.30 | 0.05 |  |  | 0.29 | 0.06 |  |
| Interaction |  |  |  | 0.48 |  |  |  | 0.43 |  |  |  | 0.73 |  |  |  | 0.86 |
| Pain-free at sleep |  | -0.23 | 0.13 |  |  | -0.24 | 0.13 |  |  | -0.01 | 0.15 |  |  | -0.04 | 0.16 |  |
| Pain-free at leisure |  | -0.05 | 0.13 |  |  | -0.04 | 0.13 |  |  | -0.09 | 0.15 |  |  | -0.07 | 0.16 |  |
| Single-site pain at sleep |  | 0.00 | 0.11 |  |  | -0.04 | 0.11 |  |  | -0.14 | 0.11 |  |  | -0.12 | 0.12 |  |
| Single-site pain at leisure |  | 0.00 | 0.11 |  |  | 0.00 | 0.11 |  |  | -0.06 | 0.11 |  |  | -0.03 | 0.12 |  |
| ln LF/HF |  |  |  |  |  |  |  |  |  |  |  |  |  |  |  |  |
| Group |  |  |  | 0.65 |  |  |  | 0.46 |  |  |  | 0.06 |  |  |  | 0.06 |
| Pain-free |  | 1.76 | 0.09 |  |  | 1.13 | 0.30 |  |  | 1.48 | 0.12 |  |  | 0.66 | 0.33 |  |
| Single-site pain |  | 1.85 | 0.07 |  |  | 1.22 | 0.29 |  |  | 1.60 | 0.08 |  |  | 0.78 | 0.32 |  |
| Multisite pain |  | 1.88 | 0.04 |  |  | 1.25 | 0.28 |  |  | 1.48 | 0.04 |  |  | 0.63 | 0.31 |  |
| Domain |  |  |  | <0.01 |  |  |  | <0.01 |  |  |  | <0.01 |  |  |  | <0.01 |
| Sleep |  | -1.41 | 0.05 |  |  | -1.40 | 0.05 |  |  | -1.63 | 0.05 |  |  | -1.64 | 0.05 |  |
| Leisure |  | -0.18 | 0.05 |  |  | -0.18 | 0.05 |  |  | -0.35 | 0.05 |  |  | -0.35 | 0.05 |  |
| Interaction |  |  |  | 0.10 |  |  |  | 0.11 |  |  |  | 0.87 |  |  |  | 0.95 |
| Pain-free at sleep |  | 0.20 | 0.11 |  |  | 0.19 | 0.11 |  |  | 0.05 | 0.13 |  |  | 0.07 | 0.14 |  |
| Pain-free at leisure |  | 0.05 | 0.11 |  |  | 0.04 | 0.11 |  |  | 0.05 | 0.13 |  |  | 0.02 | 0.14 |  |
| Single-site pain at sleep |  | -0.12 | 0.09 |  |  | -0.14 | 0.09 |  |  | 0.09 | 0.09 |  |  | 0.07 | 0.10 |  |
| Single-site pain at leisure |  | -0.01 | 0.09 |  |  | 0.00 | 0.09 |  |  | 0.08 | 0.09 |  |  | 0.05 | 0.10 |  |

IBI: interbeat intervals; SDNN: standard deviation of RR intervals; ln: natural logarithm; RMSSD: square root of the mean squared differences of successive RR intervals; LF: low-frequency power; HF: high-frequency power; LF/HF: sympathovagal balance. *Adjusted for: sex, age, BMI, smoking, moderate to vigorous physical activity at work and leisure, sitting time at work and leisure. Work domain was regarded as the reference.

**Figure S1**. Original distribution of the HRV indices during work.

**Figure S2.** Original distribution of the HRV indices during leisure.

**Figure S3.** Original distribution of the HRV indices during sleep.
